# Bone Loss, Osteoporosis, and Skeletal Fragility: Hidden Consequences of Cancer Cachexia

**DOI:** 10.3390/cells15151397

**Published:** 2026-08-01

**Authors:** Kyle H. Richardson, Jessica E. Parker, Fabrizio Pin

**Affiliations:** 1 School of Medicine, Indiana University, Indianapolis, IN 46202, USA; richarkh@iu.edu; 2 Department of Anatomy, Cell Biology and Physiology, School of Medicine, Indiana University, Indianapolis, IN 46202, USA; 3 Department of Obstetrics & Gynecology, Division of Gynecologic Oncology, School of Medicine, Indianapolis, IN 46202, USA; jesepark@iu.edu; 4 Bren Simon Comprehensive Cancer Center, Indiana University, Indianapolis, IN 46202, USA; 5 Indiana Center for Musculoskeletal Health, School of Medicine, Indiana University, Indianapolis, IN 46202, USA

**Keywords:** bone loss, BMD, osteoporosis, fracture risk, cancer cachexia

## Abstract

Cancer cachexia (CC) is a devastating, multi-organ syndrome historically defined by the progressive wasting of skeletal muscle and adipose tissue. However, emerging evidence suggests that the skeletal system is also a major, yet underappreciated target of this catabolic state. While bone loss in oncology is primarily attributed to skeletal metastasis or cancer treatment-induced bone loss (CTIBL), clinical and preclinical data has increasingly demonstrated that tumor and host-derived systemic signals can drive severe bone deterioration even in non-metastatic disease. This review synthesizes current information available on bone loss, osteopenia, and skeletal decline in the context of CC, with a specific focus on non-metastatic disease. By examining the available literature, we argue that bone loss is a “hidden” yet fundamental systemic manifestation of the cachectic state. Furthermore, we aim to highlight how concurrent muscle and bone deterioration (osteosarcopenia) dramatically worsens prognosis in several different cancers in both the adult and pediatric populations. Ultimately, this review highlights the potential contribution of cancer cachexia to osteoporosis and skeletal fragility, supporting the need for greater clinical awareness, improved musculoskeletal screening, and the development of targeted therapeutic strategies.

## 1. Introduction

Cancer cachexia (CC) is a multifactorial and complex syndrome characterized by ongoing loss of skeletal muscle mass, typically accompanied by adipose tissue wasting, that cannot be reversed by nutritional support [1]. Cachexia reflects a persistent catabolic state and negative energy balance caused by anorexia, increased energy expenditure, and profound metabolic reprogramming [2,3,4]. Depending on tumor type and disease stage, CC affects nearly half of all cancer patients and up to 80% of those with advanced-stage disease [5,6,7]. It contributes directly to 20–30% of cancer-related deaths and significantly worsens treatment tolerance, functional capacity, quality of life (QoL) and increases mortality [3,5]. Skeletal muscle loss dominates the field of cachexia research. Low muscle mass and sarcopenia are well known to be associated with higher mortality risk, increased chemotherapy toxicity, and reduced QoL in cancer patients [8,9,10,11]. However, converging evidence defines cachexia as a multi-organ syndrome. Besides skeletal muscle and white adipose tissue, cachexia involves tissues and organs such as liver, heart, brain, gut, immune system and bone, disrupting energy metabolism, endocrine signaling, and immune homeostasis across the whole organism [4,5,12,13]. These organs are now recognized as active endocrine tissues that communicate through cytokines, factors, and metabolites, creating inter-organ signaling networks that amplify inflammation and tissue wasting, thereby driving cachexia progression [14]. Clinical and preclinical evidence indicates that tumor- and host-derived signals can induce bone loss, osteoporosis, and skeletal deterioration even in the absence of bone metastases, supporting the concept that bone loss represents a systemic manifestation of the cachectic state, similar to muscle atrophy, rather than merely a consequence of local tumor invasion [15,16,17,18]. Non-bone-metastatic cancers, such as colorectal, lung, ovarian, head and neck and others, have been associated with reduced bone mineral density (BMD) and altered bone microarchitecture [16,17,19,20]. In murine models of non-metastatic cachexia, tumors drive trabecular and cortical bone loss, osteoclast activation and osteocyte damage alongside muscle wasting [15,17]. Nearly all cancer patients experience detrimental effects on the skeletal system. In this context, it has been demonstrated that BMD is reduced in cancer patients, regardless of sex or cancer type [21]. Furthermore, muscle atrophy and weakness as well as bone loss and increased risk of bone fracture have been reported to persist for months or even years after cancer remission leading to dramatically decreased QoL and poorer outcomes among survivors [22,23,24]. Bone loss in cancer patients results from both cancer treatments and the disease itself. In particular, cancer-induced systemic chronic inflammation can directly promote bone resorption, reduce bone formation, and overall impair skeletal integrity [25]. These data indicate that bone may be subjected to similar systemic mechanisms that drive skeletal muscle wasting, mediated in part by circulating tumor- and host-derived cytokines and other cachexia-associated factors (e.g., IL-6, TNF-α, RANKL, PTHrP, TGF-β, Sclerostin, Activin) [15,17,26,27,28,29].

Building on this evolving concept of cachexia as a systemic, musculoskeletal (MSK) disease, this review will synthesize current knowledge on bone loss, osteoporosis, and skeletal deterioration in cancer and cachectic cancer patients and in preclinical cachexia models, with a specific focus on non-metastatic disease. By bringing together clinical observations and mechanistic studies in animal models, and highlighting tumor–muscle–bone crosstalk, the review aims to argue that bone involvement in cachexia represents a “hidden” component of the syndrome, potentially the tip of a much larger problem, with important implications for fracture risk, survivorship, QoL and the design of MSK-targeted interventions. Unfortunately, the literature addressing bone deterioration in the absence of bone metastases remains limited, a gap that is evident in both clinical and preclinical studies. This may partly reflect the assumption that, without metastatic involvement, the skeleton is protected from cancer-induced damage, or that cancer itself, without chemotherapy, is insufficient to significantly impair bone health. In addition, bone evaluation is not routinely performed in all cancer patients. Current DEXA-based osteoporosis screening is primarily recommended for non-metastatic patients considered at elevated fracture risk [25]. Unlike weight loss and muscle wasting, which are more readily recognized during clinical evaluation, bone deterioration is often clinically silent and typically investigated only when skeletal metastases or cancer treatment-induced bone loss (CTIBL) are suspected. Consequently, cachexia-associated bone alterations occurring in the absence of bone metastases are likely underdiagnosed and underestimated.

What screening guidelines are currently in place for cancer patients without bone metastases?

Several guidelines from the American Society of Clinical Oncology (ASCO) and the National Comprehensive Cancer Network (NCCN) currently recommend active monitoring of bone loss in certain cancer survivors [25,30]. Many of the treatment modalities associated with cancer-related bone loss are commonly used in patients with breast, prostate, non-small cell lung cancer, and B-cell lymphoma, malignancies that frequently metastasize to bone and whose skeletal complications have been extensively studied. However, emerging evidence suggests that significant reductions in BMD and increased fracture risk can also occur in cancers that do not typically involve these therapies, indicating that cancer itself directly contributes to skeletal deterioration [25]. ASCO guidelines provide a broad framework for the evaluation of patients with non-metastatic disease based on established clinical risk factors [25]. These include lifestyle-related conditions such as impaired mobility, smoking, increased risk of falls, low body weight, and postmenopausal status. Risk assessment can be further complemented by the FRAX tool, which estimates a patient’s 10-year probability of major osteoporotic and hip fractures using variables such as age, sex, BMI, prior fractures, smoking history, and secondary osteoporosis. However, existing tools to evaluate risks of osteoporotic fracture have not been validated in patients with cancer [27]. Importantly, patients exposed to cancer therapies should be classified as having “secondary osteoporosis” to avoid underestimating fracture risk. Currently, dual-energy X-ray absorptiometry (DEXA) remains the recommended modality for assessing BMD [25].

One important question raised by this review is whether CC should be considered an independent risk factor for cancer-associated osteoporosis and fracture susceptibility. This hypothesis is supported by several hallmarks of CC, including body weight loss, reduced food intake, malabsorption, systemic inflammation, weakness, and reduced physical activity, all of which are known contributors to bone loss [31,32,33]. Malnutrition, for instance, promotes osteoporotic fractures not only through reductions in bone mass but also through impairment of muscle function [31]. Moreover, muscle weakness and the consequent reduction in mobility and mechanical loading can further exacerbate both muscle atrophy and bone loss [25,33]. Together, these observations support the hypothesis that bone loss, similarly to muscle wasting, may represent a systemic CC feature. Nevertheless, despite these conceptual links, direct preclinical and clinical evidence establishing a clear connection between cachexia and cancer-associated bone deterioration remains limited.

## 2. Evidence of Bone Loss in Cachexia and Cancer Patients with Non-Bone Metastatic Disease

The first evidence describing the concurrent reduction in muscle and bone mass in cachectic cancer patients was reported in 1987 by Tom Preston and Kenneth Fearon [34]. In this study, the body composition of lung cancer patients who had lost 30% of their initial body weight was compared with that of matched cancer patients with stable body weight. The study highlighted that, together with a dramatic reduction in muscle mass, there was also a significant reduction in mineral content [34,35]. This provided early evidence that the catabolic processes that drive CC extend their effects beyond muscle and adipose tissue, and into bone. Subsequent clinical studies and preclinical work have confirmed that CC is indeed associated with significant bone loss. Table 1 summarizes the evidence of bone loss reported in clinical studies of cachectic and cancer patients.

These findings are particularly relevant to gastrointestinal (GI) cancers, where CC is highly prevalent and contributes substantially to disease burden. Cachexia occurs most frequently in pancreatic, esophageal, gastric, and hepatic malignancies and is also commonly observed in colorectal cancer, although at a lower prevalence than in other GI tumor types [57,58]. Among these malignancies, pancreatic cancer has the highest incidence of cachexia, affecting up to 70–80% of patients and representing the greatest prevalence of cachexia across all cancer types [59,60]. A retrospective study demonstrated that patients with pancreatic ductal adenocarcinoma (PDAC), in addition to the classical manifestations of cachexia including low body mass index (BMI), body weight loss, and reduced skeletal muscle index (SMI) at the L3 vertebral level, also exhibited significantly lower BMD compared with control individuals [45]. Consistent with these findings, patients with PDAC were more likely to present signs of bone deterioration and osteopenia [45], defined as reduced BMD that is below normal levels but does not yet meet the diagnostic criteria for osteoporosis [61]. Furthermore, lumbar vertebral radiodensity (LVR) was positively correlated with SMI, suggesting the existence of concurrent mechanisms driving MSK deterioration in PDAC [45]. Importantly, osteopenic patients exhibited significantly reduced disease-free survival compared with controls. Moreover, osteopenia emerged as a stronger clinical predictor of disease-specific mortality than myopenia following curative surgery, suggesting the potential clinical importance of bone health assessment and therapeutic intervention in CC [45]. Similarly, a large retrospective cohort study including 1690 patients reported that individuals with PDAC experienced progressive loss of skeletal muscle area (SMA) and visceral adipose tissue as early as six months before diagnosis, with osteopenia also commonly observed in this population [36]. Another study reported that patients undergoing pancreatectomy who presented with a low cachexia index (CXI) or osteopenia had significantly poorer 5-year overall survival (OS), with CXI identified as an independent prognostic factor in pancreatic cancer. Notably, the combination of low CXI and osteopenia was associated with the worst prognosis, and the combined assessment of these parameters demonstrated greater predictive accuracy for 5-year OS than either parameter alone [48]. A retrospective study of 281 patients undergoing liver resection for colorectal liver metastases found that low preoperative BMD was associated with significantly poorer overall survival and recurrence-free survival. After adjustment for baseline differences, low BMD remained an independent predictor of adverse OS, suggesting that osteopenia may serve as a prognostic marker also in patients with colorectal liver metastases [54]. Consistent with all those findings, a meta-analysis of 23 studies found that osteopenia and osteosarcopenia, defined as the concurrent occurrence of osteopenia or osteoporosis and sarcopenia [62], were significantly associated with poorer OS and increased recurrence in GI cancers, supporting their role as important prognostic markers [53]. However, not all studies have reported consistent findings. Indeed a separate investigation found no significant association between preoperative BMD and postoperative outcomes following pancreatic tumor resection, concluding that BMD may not represent a reliable prognostic marker of tumor progression or OS in that specific cohort [49]. A systematic review including a cohort of 2230 patients affected by colorectal, esophageal, hepatic, bile duct, and pancreatic cancer reported similar findings [63]. Investigating the prognostic value of preoperative BMD on patients’ OS and recurrence-free survival (RFS), it was found that low preoperative BMD served as a significant risk factor for worse OS in multiple digestive tract cancers. Interestingly, they found that low preoperative BMD for pancreatic cancer was not a significant risk factor for OS. Similarly to OS, low preoperative BMD in colon, colorectal liver metastasis, esophageal, and extra-hepatic cholangiocarcinoma were significant risk factors for lower RFS. Osteopenia was found to influence prognosis independently of sarcopenia, suggesting it may represent an early adverse prognostic marker that precedes the development of sarcopenia in patients with digestive tract cancers [63]. Esophageal cancer (EC) is the seventh most common malignancy worldwide [64]. Treatment for EC typically involves esophagectomy, a highly invasive procedure associated with a high incidence of postoperative complications [65] and eating disorders, which further increase the risk of cachexia [66]. Previous studies have shown that preoperative sarcopenia is associated with worse clinical outcomes, and osteopenia has also been linked to sarcopenia [67]. Patients with EC frequently experience cachexia and osteopenia, and these conditions, particularly when present preoperatively, have been associated with poor prognosis following esophagectomy [68]. In one study, EC patients were stratified into four groups: cachexia, osteopenia, osteocachexia, and non-frailty. The analysis demonstrated that the cachexia group had significantly worse OS and RFS compared with the non-frailty group, whereas osteopenia alone was not significantly associated with reduced survival outcomes. However, when muscle and bone deterioration coexisted (osteocachexia), both OS and RFS were markedly reduced compared with the non-frailty group, suggesting that the combination of low preoperative CXI and osteopenia is strongly associated with adverse prognosis in patients undergoing curative esophagectomy [68]. Notably, the utility of osteopenia, sarcopenia, or osteosarcopenia as a prognostic marker extends beyond GI malignancies. Broader meta-analytic data confirm that osteopenia and osteosarcopenia serve as independent predictors of worse disease-free survival (DFS) across patients with various cancer types [14]. Both conditions are associated with an elevated risk of cancer recurrence or progression. Specifically, patients with osteopenia exhibit a 1.7-fold increase in risk (95% CI = 1.16–2.47) compared to those without, while this risk escalates to 2.17-fold (95% CI = 1.62–2.92) in osteosarcopenia.

Beyond gastrointestinal malignancies, other cancer types have also been associated with bone loss in the absence of bone metastases. Patients with head and neck cancer (HNC) are at particularly high risk of developing cachexia, with one of the highest incidences among all malignancies [3,69]. In these patients, dysphagia caused by oral cavity tumors can markedly impair food intake, contributing to cachexia and substantial involuntary weight loss, often exceeding 10% of initial body weight [70]. A study in patients with squamous cell head and neck cancer (SCHNC) demonstrated elevated serum levels of C-terminal cross-linking telopeptide of type I collagen (ICTP), a marker of bone resorption, consistent with reduced BMD at the lumbar spine, femoral neck, and hip compared with healthy controls. Bone loss was more pronounced in advanced-stage disease and appeared to be independent of smoking, alcohol consumption, and chemotherapy exposure, suggesting that SCHNC itself contributes to altered bone remodeling and reduced bone mass [20]. However, this study did not account for other parameters such as body weight or SMI, and the presence of cachexia in these patients’ cohort remains unknown.

Lung cancer is one of the most frequently diagnosed cancer types in both female and male patients and strongly associated with cachexia [71,72]. Osteodeficiency, defined as inadequate bone formation, mineralization, density, or strength, affects about half of patients with a lung cancer diagnosis. The prevalence of osteoporotic fractures in this population reaches as high as 30–40% [41]. Lung cancer is commonly known to metastasize bone, making the relationship between the two seem obvious [73]. However, there is evidence to suggest osteodeficiencies occur prior to bony metastasis. A study of 32 untreated patients with non-metastatic lung cancer, in which cachexia status was not assessed, revealed significant alterations in bone metabolism. These changes were characterized by increased levels of bone turnover markers, including osteopontin, osteocalcin, and alkaline phosphatase, as well as altered serum mineral profiles, including reduced calcium and magnesium levels [41]. These changes were associated with osteopenia and osteoporosis in a substantial proportion of patients and correlated with tumor stage, patient characteristics, and bone densitometry findings. The results indicate that lung cancer is linked to early and systemic alterations in bone metabolism, supporting the need for strategies to prevent and manage cancer-associated bone loss [41]. A multicenter study of 300 small cell lung cancer (SCLC) patients treated with immune checkpoint inhibitors found that a decrease in BMD was associated with significantly shorter PFS and OS. BMD emerged as an independent prognostic factor for OS, and its inclusion in a predictive nomogram, which was based on independent prognostic factors with the use of Cox proportional hazards analysis, improved risk stratification and survival prediction accuracy. Overall, reduced BMD over time was linked to poorer clinical outcomes in SCLC patients receiving immunotherapy [47]. Unfortunately, in these studies, CC was not investigated. Only one cross-sectional study including 52 cancer patients with lung cancer (73%) and GI tumors (15%) examined bone deterioration in association with cachexia [44]. This work shows that cachectic cancer patients have significantly higher levels of carboxy-terminal collagen crosslinks (CTX), a marker of bone resorption, while bone formation markers such as osteocalcin and procollagen type I N-propeptide (PINP) remain unchanged. However, the CTX/osteocalcin and CTX/PINP ratios indicate increased bone resorption. Hypoalbuminemia and elevated CRP are independent predictors of this imbalance, linking CC to increased bone turnover driven by inflammation and poor nutritional status [44].

Sex-specific cancers, including breast, prostate, cervical, ovarian, and endometrial cancers, are malignancies that are frequently treated with agents known to compromise bone health (e.g., androgen deprivation therapy, aromatase inhibitors). The use of these treatments automatically categorizes you as having secondary osteoporosis with tools like the FRAX calculator, thus increasing a patient’s 10-year probability of a fracture. However, it should be noted that these cancers also show signs of decreased BMD in the absence of bone metastasis or adverse treatment effects. In prostate cancer, it is known that androgen deprivation therapy (ADT) can cause bone loss, and the percentage of osteoporosis in men treated with ADT for prostate cancer is as high as 53% [40]. To determine the bone health of patients who were “hormone naïve” or had never received ADT but still had a diagnosis of prostate cancer, a systematic review of the literature found significant bone loss in 80% of “hormone naïve” prostate cancer patients. Of the 80%, they estimate 3.9–37.8% had osteoporosis. The papers in this review quantified bone loss using DEXA from mainly the lumbar spine or hip. Other findings of note include that most men in the ADT group had been diagnosed with metastatic disease compared to the hormone naïve group. Overall, they found that those in the ADT group had significantly worse bones than hormone naïve, with hormone naïve patients still having significantly worse BMD than age-matched healthy individuals. This highlights that men with prostate cancer are at risk for osteoporosis and a theoretical risk of increased fractures regardless of therapy [40]. Like prostate cancer, breast cancer also commonly metastasizes to bone, leading to decreased bone mineral density, especially in areas of metastasis. Investigations into the fracture risk in patients with non-metastatic breast cancer reveal a high incidence of vertebral fracture even without evidence of skeletal metastasis [38]. At the time of initial diagnosis, researchers did not find a significant difference in BMD when compared to healthy age-matched individuals. However, in the three years following diagnosis, the incidence of vertebral fracture was nearly five times higher than the normal population. Notably, the vertebral fracture risk increased 20-fold in patients with recurrent soft tissue breast cancer without evidence of skeletal metastasis. The researchers hypothesized that the actual incidence of fracture in this study is perhaps underestimated as participants received varying doses of clodronate [38]. The temporal relationship of BMD changes and increased fracture risk highlights the need for continued surveillance even after treatment is completed. Rates of cervical cancer have largely been reduced with medical advances, namely the HPV vaccine. However, cervical cancer is still common, and there are great proactive guidelines in place to help detect cervical cancer. Like breast and prostate cancer, cervical cancer can metastasize to bone. While the most common site of metastasis in patients with cervical cancer is the lung, the second most common site is bone [74]. For instance, a prospective study using DEXA scans found that patients with invasive cervical cancer without bone metastases had significantly lower BMD than age-, BMI-, and weight-matched healthy controls [16]. Interestingly, they did not observe significant differences in the levels of calcium or phosphate in the blood. With the significantly lower BMD, they again hypothesized that this patient demographic is at an increased risk of developing osteoporosis [16]. Additionally, observations of a specific subgroup of cervical cancer patients found overall lower BMD in patients with invasive cervical cancer when compared to age-matched women. Noticeably, the only significant difference in BMD was noted in women in their fifties, less than 5 years after menopause [37]. Gynecological cancers are often associated with bone loss, partly due to treatment-related effects. However, the most common gynecologic cancers, endometrial and ovarian, are not commonly associated with bony metastases. Despite this fact, in a study evaluating bone health before and after remission, patients with ovarian cancer had lower BMD than controls even before treatment. Following treatment, uterine cancer patients experienced the most rapid decline in BMD. Bone loss varied by treatment modality, with the smallest reductions observed in patients treated with surgery alone and the greatest losses in those receiving postoperative chemotherapy. These decreases were most pronounced at the lumbar spine (L3–L4) and femoral neck [42]. Unfortunately, in studies focused on sex-specific cancers, cachexia was not taken into account. Therefore, it remains unclear whether cachexia contributes to the observed bone loss in these cancers. Since several of these cancer types have a high prevalence of cachexia and are also associated with skeletal alterations, further studies are needed to determine whether cachexia and bone deterioration develop concurrently and share common underlying mechanisms.

## 3. Long-Term Bone Alteration in Adults and Children Cancer Survivors

Much of what has been discussed thus far refers to changes observed in patients with active cancer. However, emerging evidence suggests that these skeletal alterations may persist long after tumor remission. Previous studies show that the MSK system of cancer survivors often does not fully recover, as evidenced by persistent frailty and MSK decline, including muscle atrophy, weakness, as well as bone loss and an increased risk of fractures. Together, these alterations reduce QoL and survival in cancer patients [22,75,76]. Unfortunately, similar MSK impairments are also prevalent in childhood cancer survivors [77,78].

Highlighting this population-level risk, a large UK study looked at survivors of 20 of the most common cancers in their population with the goal of assessing bone and any major osteoporotic fractures. They found evidence of increased bone fractures in cancer survivors of 15/20 of the most common cancer types [22]. They also found that the major osteoporotic fracture risk increased in 17/20 cancers. For some of these cancer types, the hazard ratio (HR) for the fracture risk tapered after 5 years, but persisted beyond 5 years [22]. Interestingly research has suggested that different types of cancer increase risk of different types of fractures. An analysis from Ontario found that breast cancer was associated with fracture risk in all measured sites, whereas colorectal cancer was associated with hip and femur fractures, but not spine or pelvis. This study also found results like the larger UK study in that patients with cancer are at “a modest risk” of fractures compared to the non-cancer controls, notably in breast, prostate, lymphoma, and multiple myeloma cancers [46]. Amongst breast cancer patients, similar results have been observed. One study compared the BMD of post-menopausal breast cancer survivors to those of post-menopausal women who have never had breast cancer. Participants were non-users of hormone therapy or tamoxifen therapy in the prior six months. They found that the presence of lower BMD was higher in post-menopausal breast cancer survivors compared to post-menopausal women without breast cancer [39]. Comparable findings are present in younger breast cancer survivors, where incident osteopenia and osteoporosis were significantly higher within a few years of diagnosis than in cancer-free women, though risk varies by treatment [56]. Women in this study with ER-positive breast cancer were observed as having a two-fold increase in the risk of osteopenia and osteoporosis, which is more likely due to hormone therapy than to differences in the tumors themselves. Notably, survivors diagnosed at age ≤ 50 had almost a two-fold increased risk (95% CI = 1.21–3.24) of osteopenia and osteoporosis as well [56]. ADT as well as radiation therapy are commonly used, often together, in the treatment of prostate cancer. When evaluating whether CT measured BMD and muscle mass in prostate cancer patients after treatment has concluded, results showed that psoas muscle mass and L5 BMD were independent predictors of noncancer death in men with a history of prostate cancer [55]. Even though prostate cancer has a high incidence, the estimated ten-year prostate cancer mortality has been consistently less than 10%. These parameters being independent predictors of non-cancer death is huge, especially since the mortality rate is lower for the disease itself, meaning side effects of treatment potentially carry the majority of risks.

To understand the effects of treatment and certain cancers on the bone health of pediatric patients, a comprehensive meta-analysis of BMD in childhood cancer survivors (CCS) looked at hematologic, solid/central nervous system tumors, as well as mixed tumors. They found that via pooled Z-scores, which are age and sex-appropriate standard deviations, CCS had significantly reduced BMD at all skeletal sites measured compared to age-matched controls [51]. Another similar systematic review of CCS and the incidence of osteoporosis found similar results, showing an overall decline in BMD in children undergoing cancer treatments, which again, persists into adulthood. Interestingly, in this study, they found that children undergoing cancer treatment had a more pronounced decrease in BMD at the hip/femoral neck compared to the lumbar spine [52]. This study also created a pooled fracture prevalence among children undergoing cancer treatment, and it was 13%. Validating these findings, another study estimated the prevalence of low BMD in CCS to be anywhere from 9 to 51% [43]. Moreover, 6 years after cancer diagnosis younger cancer survivors were at increased risk for low lumbar BMD [79]. Sarcopenia has also been shown to coincide with osteopenia. In one study looking at CCS, they found significantly lower aBMD z-scores in the sarcopenia group when compared to the others. aBMD scores were calculated by the bone mineral content (BMC) divided by the two-dimensional area of the bone [80]. Their study found that the majority had hematologic cancer, and males presented with higher percentages of sarcopenia [50].

## 4. Bone Deterioration in Preclinical Mouse Models of Cancer Cachexia

Much of what is known about bone deterioration in CC has emerged from preclinical murine models, which have proven indispensable in demonstrating that non-metastatic tumor growth can drive significant skeletal deterioration independent of both direct bone colonization and cancer treatment. These alterations in bone, similar to muscle wasting, are driven by systemic signals. However, the underlying pathological mechanisms remain poorly understood. This gap in knowledge is partly attributable to the limited availability of preclinical models that recapitulate bone loss in the absence of bone metastases. Moreover, few studies have evaluated sex differences, with the vast majority conducted in male mice. Most studies have also relied on actively growing young mice rather than adult mice, which better reflect the human cancer population, and have used rapidly growing tumors with short experimental durations. Finally, cancer cachexia-associated bone deterioration has only recently emerged as a major area of investigation.

We are now reviewing the published preclinical models of CC that demonstrate bone deterioration in the absence of direct bone metastasis. The key characteristics of each model, including sex, age, duration, presence of muscle wasting and bone deterioration, are summarized in Table 2.

The first study describing concurrent muscle and bone loss during CC was reported by Choi and colleagues in 2013 [81]. Using a Lewis lung carcinoma (LLC) model in which tumor cells were injected into the hindlimb muscle of 8-week-old male mice, the authors demonstrated that LLC growth, associated with reduced fat and lean mass, also caused decreased BMD [81]. This study provided the first evidence that bone loss occurs during CC independently of bone metastasis. Similarly, subcutaneous LLC injection into the thigh induced reduced BMD, trabecular bone volume, and trabecular thickness, with increased trabecular porosity. Importantly, these effects were more pronounced in male mice, whereas females showed a milder phenotype [82]. Consistent with these findings, our group demonstrated that subcutaneous intrascapular LLC tumors induce systemic bone deterioration, including reduced trabecular and cortical bone volume in male mice [17]. A recent publication from our group established a model of lung-derived liver metastases that recapitulates an advanced stage of lung cancer [83]. In this model, the presence of LLC liver metastases markedly accelerates the development of cachexia, as evidenced by significant muscle wasting and weakness after only 18 days of tumor burden, whereas these alterations are not observed at the same time point in the subcutaneous model [83]. Similarly, bone microstructural deterioration and impaired mechanical properties are also evident in the metastatic model, while comparable changes in the subcutaneous model emerge only after an additional 7 days of tumor burden [83]. These findings indicate that metastatic progression exacerbates the development and severity of cachexia, a clinically relevant observation considering the higher prevalence of cachexia among patients with metastatic lung cancer [89]. It has been reported that LLC cells produce and release parathyroid hormone-related protein (PTHrP) into the circulation in mice and this has been implicated in LLC-induced CC [27]. In addition, PTHrP promotes osteoclastic bone resorption and hypercalcemia [90]. Therefore, in the LLC model of cachexia, elevated circulating levels of PTHrP may contribute to both muscle wasting and bone loss.

One of the most widely studied cancers associated with cachexia is colorectal cancer (CRC), which has long been associated with severe muscle deterioration [91]. It has been shown, using three different CRC models: the C26 (Colon-26) adenocarcinoma, the HT-29 xenograft, and the C57BL/6J-Apc^Min+^ mouse model, which spontaneously develops adenomas (polyps), that cachexia is associated with varying degrees of bone deterioration across these models [84]. Apc ^Min+^ mice show the greatest reduction in total, vertebral, and femoral bone mineral density (BMD; −18%, −22%, and −31%, respectively), followed by HT-29 with intermediate reductions (−5%, −11%, and −15%), and lastly the C26 model, which shows a reduction of −8% only in total BMD. These data are consistent with similar trends observed in alterations of bone microarchitecture and mechanical properties [84]. Interestingly, the degree of muscle wasting is not always proportional to bone loss. Indeed, aside from Apc ^Min+^ mice, the C26 model, despite exhibiting a more severe cachexia phenotype than HT-29, has only a minimal impact on bone deterioration [84]. This discrepancy could be explained by differences in the tumor-derived secretome or in tumor growth kinetics; the shorter duration of the C26 model (~14 days) compared with HT-29 (~47 days) may indicate that bone resorption requires a longer time to develop than muscle wasting. It is well known that more advanced cancer stages and metastatic disease are associated with a higher degree of cachexia [92]. In this regard, our group previously developed two models to study CRC–associated liver metastasis–induced cachexia, which mimic advanced disease and are associated with exacerbated MSK deterioration [85,86]. The intrasplenic injection of C26 cells induces the development of multiple liver metastases that severely affect trabecular bone microarchitecture in the host [85]. The second model uses MC38 cells, which have been associated with divergent cachexia phenotypes, with previous studies describing both the presence and absence of cachectic features [86,93]. However, in our experiments, both the subcutaneous and metastatic variants of this model exhibited cachexia, albeit to different degrees [86]. The subcutaneous model displayed body wasting that was further exacerbated in the presence of liver metastases. Similarly, skeletal muscle wasting was markedly more pronounced in the metastatic model compared with the subcutaneous model, with muscle reductions of approximately 13% and 28%, respectively [86]. Interestingly, in this context, cachexia severity does not appear to be associated with a proportional extent of bone loss. Indeed, both models showed a similar degree of trabecular bone volume reduction (−49% and −46%) and cortical bone volume reduction (−12% and −8%) in the subcutaneous and metastatic models, respectively [86]. These observations suggest that the MSK system responds differentially to tumor burden, and that muscle and bone impairments may be regulated by partially distinct underlying factors. In contrast, another study published by our group indicates that blockade of activin receptor type 2B in the mHCT116 model protects both tissues from CRC-induced MSK deterioration, suggesting context-dependent shared mechanisms in the regulation of muscle and bone in cancer settings [28].

Ovarian cancer, historically the deadliest gynecologic malignancy, has been associated with the development of cachexia. However, muscle wasting in ovarian cancer remains underrecognized, partly because it is often masked by obesity, ascites, and abdominal distension [94], as well as by the lack of well-characterized preclinical models. Our group was the first to characterize an ovarian cancer-induced cachexia model [87]. Intraperitoneal injection of ES-2 cells, representative of clear cell carcinoma [95], induced reduced tumor-free body weight, severe muscle wasting, and myofiber atrophy [87]. This model also caused reduced BMD and BMC, decreased trabecular bone volume (~33%), and alterations in trabecular parameters in femoral and vertebral compartments [87]. MSK deterioration was associated with increased circulating Receptor Activator of Nuclear Factor κB Ligand (RANKL/Tnfsf11), an osteocyte-derived regulator of osteoclastogenesis and bone resorption [15]. We demonstrated that ES-2 cells are the primary source of RANKL in this system, further supporting the concept that tumor-derived factors are key drivers not only of muscle atrophy but also of bone resorption. Interestingly, neutralization of RANKL using anti-RANKL therapy not only preserved bone mass but also concurrently improved muscle mass and function [15]. These observations further support our hypothesis that muscle wasting and bone resorption can be regulated by a common mechanism during CC.

**Figure 1:**
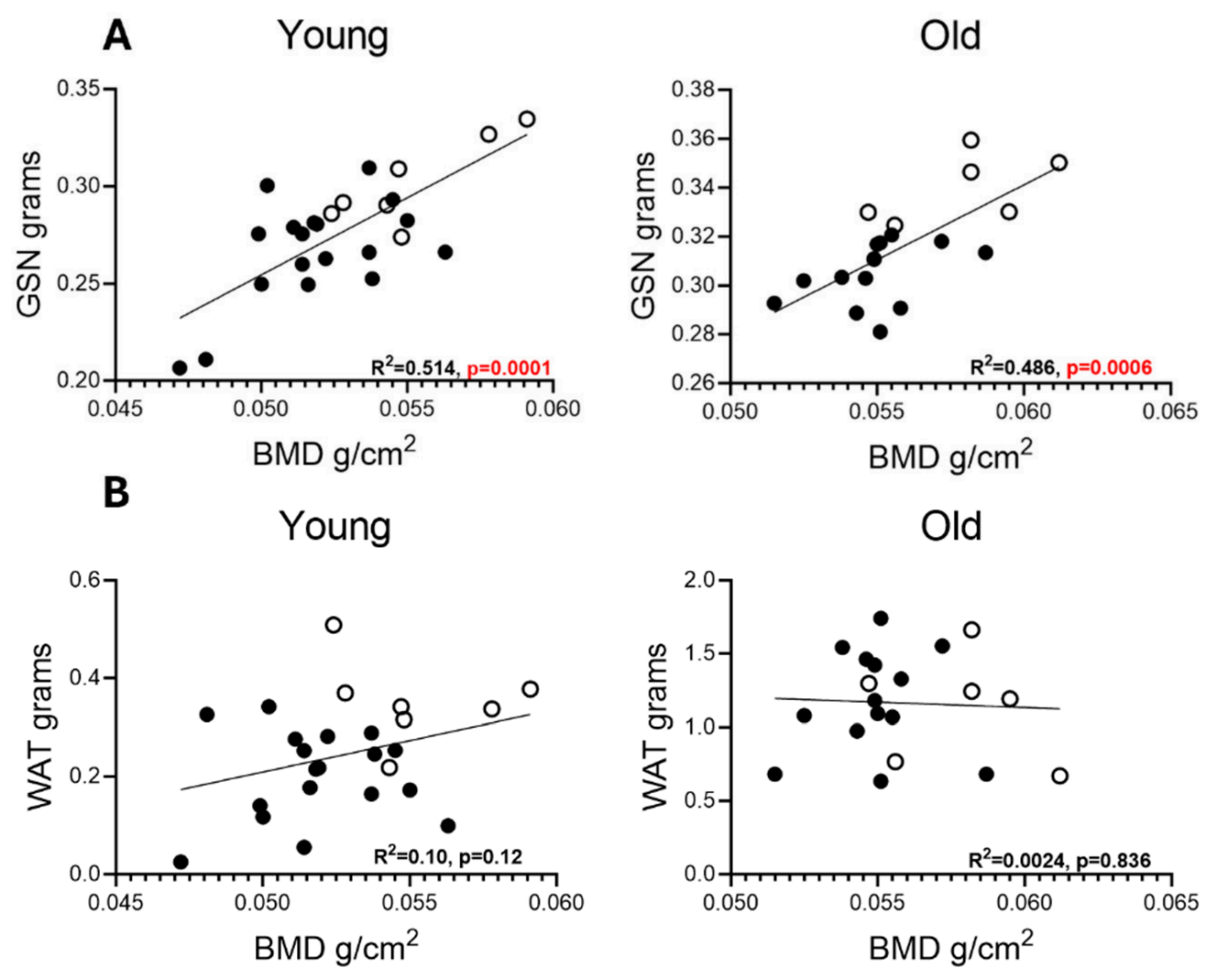
(**A**) Correlation between gastrocnemius (GSN) muscle mass (g) and bone mineral density (BMD; g/cm^2^) in young and adult male mice. (**B**) Correlation between white adipose tissue (WAT) mass (g) and BMD (g/cm^2^) in young and adult male mice. Solid black circles represent HNC-bearing mice, whereas open white circles represent control mice. Correlations were analyzed using simple linear regression. The coefficient of determination (R^2^) and the corresponding *p* value are shown on each graph.

Patients with Head and Neck Squamous Cell Carcinoma (HNSCC) are at particularly high risk of CC, with more than 40% presenting with cachexia at diagnosis or treatment initiation [69]. Although HNSCC represents a smaller proportion of cancer incidence, it contributes substantially to CC-related morbidity and mortality [96,97]. However, preclinical models to study MSK deterioration in HNSCC remain limited. Olson and colleagues characterized cachexia using HPV-positive squamous cell carcinoma MLM3 and MLM5 allograft models [98]. In young, growing male and female mice, these tumors induce marked muscle wasting and hypercatabolism [98]. We further analyzed tumor-associated bone deterioration in both young (2-month-old) and adult (12-month-old) mice, the latter better reflecting the age distribution of HNC patients [19]. MLM3 tumors induced muscle wasting, reduced BMD and BMC, impaired trabecular and cortical architecture, and decreased mechanical strength in both age groups [19]. Conversely, MLM5 primarily caused muscle loss in adult mice and affected skeletal outcomes mainly in young animals [19]. This study clearly demonstrates that HNSCC-induced MSK deterioration differs between young and adult mice, resulting in impaired growth in young animals, whereas mature mice exhibit overt MSK loss. To better understand the relationship between muscle and bone loss in these HNC models, we analyzed previously published [19] data to assess the correlation between skeletal muscle mass and BMD in young and adult mice. As shown in Figure 1, muscle mass was strongly correlated with BMD at both ages, indicating that muscle and bone deteriorate in tandem during HNC-induced cachexia. In contrast, no correlation was observed between white adipose tissue mass and BMD, despite the marked adipose tissue wasting associated with cachexia. These findings suggest that muscle and bone share common mechanisms driving MSK deterioration during CC. Another HNSCC model consisting of B0092 cells has been shown to also induce severe CC in male mice and milder effects in females, characterized by muscle wasting and weakness [88]. Notably, in this model, cachexia is also associated with significant bone loss [88]. We acknowledge that independent validation of the mechanisms underlying bone deterioration in preclinical models of cancer cachexia remains limited. This represents a current limitation of the field, highlighting the need for additional studies from independent laboratories to further validate and expand our understanding of the mechanisms involved.

## 5. Conclusions and Future Perspectives

**Figure 2:**
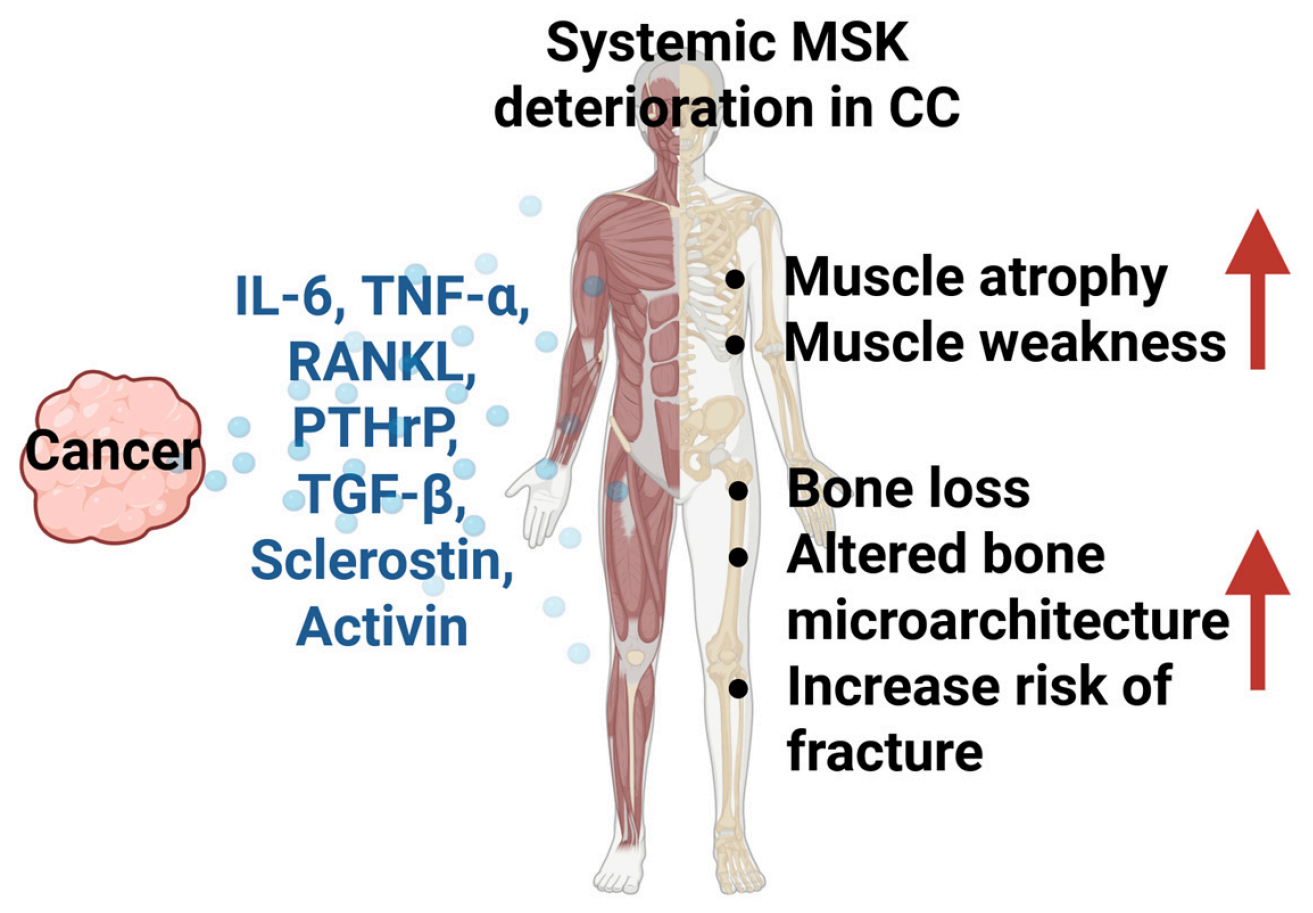
Schematic representation of the systemic drivers of musculoskeletal (MSK) deterioration associated with cancer cachexia (CC). Clinical and preclinical evidence suggests that bone loss is a systemic manifestation of the cachectic state, analogous to skeletal muscle wasting. Tumor- and host-derived factors, including IL-6, TNF-α, RANKL, PTHrP, TGF-β, sclerostin, and activin, are shown to target both skeletal muscle and bone, driving MSK deterioration that contributes to functional decline, frailty, poor clinical outcomes, and reduced quality of life in patients with CC.

Accumulating clinical and preclinical evidence demonstrates that bone deterioration is a prevalent yet underrecognized consequence of cancer and CC. Across multiple cancer types, reductions in BMD, alterations in bone microarchitecture, and increased fracture risk occur even in the absence of bone metastases and, in some cases, prior to the initiation of anticancer therapies. These skeletal alterations frequently coexist with muscle wasting and weakness, supporting the emerging concept that CC is not solely a muscle disorder but rather a systemic musculoskeletal syndrome characterized by coordinated dysfunction of both bone and muscle. These skeletal alterations may be driven by the same tumor-derived factors that simultaneously target muscle tissues, as illustrated in Figure 2. Our initial question of whether cancer cachexia may represent an independent risk factor for osteoporosis is plausible and merits further investigation. However, the current evidence remains limited, as most clinical studies are retrospective or observational, and several do not formally diagnose cachexia. Therefore, the current evidence supports discussing the broader association between cancer and bone loss rather than defining CC as an independent risk factor for osteoporosis. These observations raise important clinical considerations. *Bone deterioration may represent an important component of cancer cachexia, a possibility that warrants further investigation. Routine evaluation of bone health in patients with cachexia represents a potential area for clinical improvement. The need for broader skeletal screening in patients with cancer remains to be determined.* If bone loss is indeed an intrinsic consequence of cancer and cachexia, current screening guidelines may substantially underestimate the burden of skeletal disease in oncology patients. Addressing these questions will require prospective clinical studies integrating cachexia status, muscle mass, BMD, and fracture risk assessments. Moreover, improving preclinical models and incorporating bone health evaluations into cachexia research will be essential. Recognizing bone deterioration as a fundamental component of cancer-associated MSK dysfunction may not only redefine our understanding of cachexia but also open new opportunities for diagnosis, risk stratification, and therapeutic intervention. Ultimately, preserving both muscle and bone may prove essential for improving quality of life, treatment tolerance, survivorship, and overall outcomes in patients with cancer.

## Figures and Tables

**Table 1:** Summary of bone deterioration in clinical studies.

Author	Cancer Type	BMD Measurements	Sample Size	Study Design	Key Results
Klatte et al. 2024 [36]	PDAC	Abdominal CT	516	Retrospective cohort	Loss of muscle tissue and bone volume were seen in the last 6 months before diagnosis
Cho et al. 1991 [37]	Cervical	Dual-photon absorptiometry	233	Case–control	Mean BMD in cervical cancer was 12.8% lower than controls
Kanis et al. 1999 [38]	Breast	Nd (vertebral fracture)	352	Prospective cohort	Vertebral fracture risk was ~5x higher than normal from time of first diagnosis
Hung YC et al. 2002 [16]	Cervical	DXA	50	Prospective case–control	Spinal BMD significantly lower in cervical cancer patients vs. controls
Valero C et al. 2006 [20]	Squamous Cell Head and Neck	DXA	80	Case–control	BMD measurements were significantly lower in cancer patients vs. controls
Conde DM et al. 2012 [39]	Breast	DXA	122	Cross-sectional	Abnormal BMD significantly more frequent in breast cancer survivors vs. controls
Lassemillante AC et al. 2015 [40]	Prostate	DXA	Nd	Systematic review and meta-analysis	Osteoporosis still prevalent in hormone-naïve cancer patients
Dumanskiy YV et al. 2018 [41]	Lung	DXA	32	Cross-sectional	Osteodeficiency observed in ~50% of patients, more common in women
Lee JE et al. 2020 [42]	Gynecological	DXA	243	Retrospective cohort	Cervical and Ovarian cancer BMDs were significantly lower before treatment
Jin HY et al. 2020 [43]	Childhood cancer	DXA	Nd	Narrative review	Childhood cancer survivors have a lower BMD than the general population
Zwickl H et al. 2021 [44]	Multiple	Nd	52	Cross-sectional	Cachectic patients had significantly higher bone resorption markers
Cameron ME et al. 2022 [45]	PDAC	CT	134	Retrospective cohort	61.2% of PDAC patients were osteopenic vs. 36.8% of controls
Gong IY et al. 2023 [46]	Multiple	Nd (fracture outcomes)	172,963	Retrospective cohort	Cancer patients had higher fracture risk vs. controls
Buzasi E et al. 2024 [22]	Multiple	Nd	578,160	Matched cohort	Fracture risk increased in 15/20 cancers (17/20 for osteoporotic fracture)
Liu W et al. 2025 [47]	Small cell lung	CT	300	Retrospective cohort	Greater bone loss was independently associated with shorter OS in SCLC patients
Sakamoto T et al. 2025 [48]	Pancreatic	CT	121	Retrospective cohort	Patients with osteopenia had significantly worse 5-year OS
Sharshar M et al. 2020 [49]	Pancreatic	CT	275	Retrospective cohort	Lower preop BMD was not a risk factor for mortality
Marmol-Perez A et al. 2024 [50]	Pediatric (multiple)	DXA	116	Cross-sectional	Survivors with confirmed sarcopenia had significantly lower aBMD z-scores
Velentza L et al. 2024 [51]	Childhood (multiple)	DXA	8106	Systematic review/meta-analysis	BMD was significantly lower in survivors vs. controls at all sites
Markarian AM et al. 2025 [52]	Childhood (multiple)	DXA	4547	Systematic review/meta-analysis	BMD z-scores significantly lower in survivors at lumbar, hip, and femoral neck
Zou X et al. 2025 [53]	GI	CT	23 studies	Systematic review/meta-analysis	Osteopenia was a significant risk factor for worse OS and RFS.
Ikuta S et al. 2021 [54]	Colorectal	CT	281	Retrospective cohort	Low BMD group has significantly worse OS
McDonald AM et al. 2017 [55]	Prostate	CT	653	Retrospective cohort	Low L5 BMD was an independent predictor of noncancer death
Ramin et al. 2018 [56]	Breast	Nd	778	Prospective cohort	BC survivors had 68% higher risk of osteopenia/porosis

**Table 2:** Summary of bone deterioration in preclinical mouse models of cancer cachexia.

Author	Model	Sex	Mice Age	Duration	Cachexia	Muscle Wasting	Adipose Wasting	Reduced BMD	Altered Bone Microarchitecture	Key Results
Choi et al.; 2013 [81]	LLC (Hindlimb)	Male	8-week-old mice	21 or 25 days	Yes	Yes (at 25 days)	Yes (at 25 days)	Yes	nd	First evidence of bone loss in cancer cachexia
Berent et al.; 2020 [82]	LLC (s.c.)	Female and Mela	7-week-old mice	21 days	nd	nd	nd	Yes	Yes	Male mice exhibit greater bone loss
Pin et al.; 2021 [17]	LLC (s.c.)	Male	8-week-old mice	28 days	Yes	Yes	Yes	nd	Yes	Osteocytic osteolysis
Gonzales et al.; 2026 [83]	LLC (metastatic and s.c.)	Male	8-week-old mice	18 (metastatic) or 25 (s.c.) days	Yes	Yes	Yes	nd	Yes	Phenotype more pronounced in metastatic LLC
Bonetto et al.; 2017 [84]	C26 (s.c.)	Male	8-week-old mice	14 days	Yes	Yes	Yes	Yes	No	Minimal bone deterioration but severe Cachexia
Bonetto et al.; 2017 [84]	HT-29 (s.c.)	Male	8-week-old mice	47 days	Yes	Yes	Yes	Yes	Yes	Equal bone and muscle deterioration
Bonetto et al.; 2017 [84]	C57BL/6J-Apc^Min+^	Male	12-week-old mice	27 weeks	Yes	Yes	Yes	Yes	Yes	Equal bone and muscle deterioration
Huot et al.; 2020 [85]	C26 (metastatic	Male	12-week-old mice	15 days	Yes	Yes	Yes	nd	Yes	Metastatic CRC induces MSK deterioration
Huot at all; 2020 [86]	MC38 (metastatic and s.c.)	Male	8-week-old mice	19 (metastatic) or 28 (s.c.) days	Yes	Yes	Yes	nd	Yes	Cachexia more pronounced in metastatic MC38, but similar degree of bone deterioration
Huot at all; 2020 [28]	HCT116 (metastatic)	Male	8-week-old mice	25 days	Yes	Yes	Yes	nd	Yes	Metastatic CRC induces MSK deterioration
Pin et al.; 2018 [87]	ES-2 (i.p.)	Female	8-week-old mice	15 days	Yes	Yes	Yes	Yes	Yes	First evidence of bone and muscle loss in Ovarian CC
Jones et al.; 2025 [19]	MLM3 and MLM5 (s.c.)	Male	8-week and 12 month -old mice	26 days	Yes	Yes	Yes	Yes	Yes	HNC growth affects muscle and bone in an age-dependent manner
Livingston et al.; 2025 [88]	B0092	Female and Mela	10-week-old mice	33 days	Yes	Yes	Yes	nd	Yes	Phenotype more pronounced in male mice

nd = not determined/not assessed.

## Data Availability

No new data were created or analyzed in this study. Data sharing is not applicable to this article.
